# Use of and Satisfaction With Mobile Health Education During the COVID-19 Pandemic in Thailand: Cross-sectional Study

**DOI:** 10.2196/43639

**Published:** 2023-01-24

**Authors:** Kamonrat Kittipimpanon, Angun Noyudom, Pawanrat Panjatharakul, Poolsuk Janepanish Visudtibhan

**Affiliations:** 1 Ramathibodi School of Nursing Faculty of Medicine Ramathibodi Hospital Mahidol University Bangkok Thailand

**Keywords:** mHealth, COVID-19, chatbot, use, satisfaction

## Abstract

**Background:**

RamaCovid is a mobile health (mHealth) education system that provides the Thai population with information about COVID-19 and self-risk assessment. RamaCovid has a chatbot system that provides automatic conversations (available 24 hours per day) and a live chat function that allows users to directly communicate with health professionals (available 4 hours per day in the evening). The system consists of (1) COVID-19 vaccine information, (2) self-care after vaccination, (3) frequently asked questions, (4) self-risk assessment, (5) hospital finding, (6) contact number finding, and (7) live chat with a health professional.

**Objective:**

This study investigates the use of and satisfaction with the RamaCovid system.

**Methods:**

Overall, 400 people were recruited via RamaCovid by broadcasting an infographic about the study. Questionnaires collected demographic data, users’ experiences of RamaCovid, and the use of and satisfaction with the system. The questions were answered using a 5-point Likert scale. Descriptive statistics were used to describe the participant characteristics and their use of and satisfaction with the RamaCovid system. The Mann-Whitney *U* test was performed to examine the difference in use and satisfaction between the adult and older adult groups.

**Results:**

The participants showed high use of and satisfaction with the RamaCovid system. They used the information to take care of themselves and their family, and they gained information about their COVID-19 risk. The users were satisfied with the system because the information was easy to understand, trustworthy, and up to date. However, the older adult group had lower use of and satisfaction with the system compared to the adult group.

**Conclusions:**

RamaCovid is an example of the successful implementation of mHealth education. It was an alternative way to work with the call center during the COVID-19 pandemic and increased access to health information and health care services. Providing ongoing updated information, improving the attractiveness of the media information, and the age group difference are important issues for further system development.

## Introduction

### Background

COVID-19 is a communicable disease of the respiratory system and is caused by infection with SARS-CoV-2. This virus was first detected in Wuhan, Hubei Province, China, in December 2019 [1]. COVID-19 rapidly spread to several countries, and by March 31, 2020, approximately 172 countries reported a total of nearly 750,000 cases and over 3300 deaths [2]. As of April 3, 2022, more than 489 million cases and more than 6 million deaths have been reported worldwide [3]. The first confirmed case of COVID-19 in Thailand was a Chinese tourist in Bangkok and was identified on January 13, 2020 [4]. The first Thai COVID-19 patient was identified on January 31, 2020. The initial wave of infection occurred in March 2020 and stemmed from an outbreak of clusters related to Boxing Stadium; this outbreak caused widespread disease and continuously increasing numbers of patients [5]. The COVID-19 vaccination program in Thailand was launched in March 2021 with 2 types of vaccine platforms, an inactivated virus vaccine (SINOVAC) and a viral vector vaccine (Oxford-AstraZeneca). Health care professionals were the first to receive vaccination. By May 2021, older adults and high-risk populations were able to reserve a vaccine appointment via an official COVID-19 vaccine app, with mass vaccinations beginning in June 2021.

COVID-19 is an emerging disease that can be transmitted to and between humans through contact with secretions. It is spread through small and large droplets that carry the virus and enter the body through the eyes, nose, and mouth [6]. The most common symptoms are fever, cough, sneezing, shortness of breath, and fatigue, but severe acute respiratory distress can also occur [7,8]. The incubation period of the virus is approximately 2-14 days, and the virus can be transmitted by asymptomatic individuals [9]. Thai people were aware of and concerned about COVID-19, and they needed information about this disease, such as how to recognize symptoms, information about its transmission, how to conduct self-assessment, how to access COVID-19 testing (reverse transcription polymerase chain reaction [RT-PCR]), and disease prevention techniques. In addition, when the COVID-19 vaccination program was launched, some people were hesitant or refused immunization because they were concerned about the safety and potential side effects of the vaccines. People needed information about the vaccines to help them make informed vaccination decisions.

Hotlines connected to call centers have been essential in pandemic situations and emergencies because they provide a communication channel for people to access up-to-date information. Hotlines are easily accessible, and they reduce exposure and disease spread. Call centers can also constitute a multichannel communication system and conduct telephone triage to reduce travel and congestion in hospitals. They can provide advice and information about COVID-19 prevention behaviors and inform people about how to conduct self-assessment [10]. In Thailand, a COVID-19 call center associated with the Ramathibodi School of Nursing was established in March 2020. The call center workers provided information about COVID-19 triage by communicating guidelines on clinical practice, diagnosis, treatment, and the prevention of health care–associated COVID-19 infection [11], as well as providing general COVID-19 education. The aim was to enhance people’s knowledge and understanding and relieve doubts and anxiety about COVID-19. Frequently asked questions were primarily about self-risk assessment, disease information and treatment, and prevention behaviors. However, there was limited service time; thus, many people could not access this service. Digital health or mobile health (mHealth) is an alternative way to increase communication in accordance with current needs.

Digital health, specifically mHealth, is defined as the use of mobile devices and other communication technologies to improve the quality of health care and the coverage of care by increasing access to health information services [12,13]. Mobile technologies are capable of multifaceted functions through the rapid dissemination of information, allowing for timely risk stratification and providing alerts. They can also provide easy access to institutional protocols and guidelines, be used in early diagnosis, help identify transmission paths, and promote coping behaviors in the general population [13]. Mobile technology disseminated up-to-date and validated information about SARS-CoV-2 for health care professionals [14].

Today, most people can access a mobile telephone daily, and mobile phones are often used for social communication, searching for information, and entertainment. The International Telecommunication Union reports that over than 7 billion people use mobile telephones. In 2021, an estimated 4.9 billion people used the internet, or roughly 63% of the world’s population [15]. In Thailand, approximately 50.1 million people use the internet, which is more than 75% of the total population (66.5 million people), and 95.3% of the users use the internet for social media communication via apps, such as Facebook, Line, and Instagram. Line is the most popular online communication channel in Thailand and is used by 98.5% of Thai internet users (approximately 47 million people) [16]. Thus, mHealth that is accessed via Line should be developed to respond to and communicate with the public during the COVID-19 pandemic in Thailand.

### RamaCovid

RamaCovid is a Line Official Account (Line OA) belonging to the Line app. It was developed using information recorded by the COVID-19 call center service in accordance with frequently asked questions. The team designed and trained a chatbot system that could carry out automatic chat conversations. The information provided by the chatbot was validated by infectious disease specialists and was updated following the Ministry of Public Health guidelines before the chatbot became available to the general public. Information is provided in the form of text messages, infographics, links, telephone numbers, and the Global Positioning System (GPS) locations of hospitals. This system can be accessed 24 hours a day. RamaCovid also provides a live chat function, where health professionals directly talk with people who have complex questions; this service is available for 4 hours per day in the evening (4:00-8:00 p.m.). The first version of RamaCovid was launched on March 11, 2021. Approximately 11,726 people initially added friends in the RamaCovid Line OA. At present, it reaches up to 63,000 people. Many people use the system daily to find information, with approximately 3129-11,030 users per month (mean 5414, SD 2284.05), depending on the COVID-19 situation. Use and satisfaction are critical indicators that can help improve the system. Thus, we examined people’s use of and satisfaction with the RamaCovid system.

## Methods

### Participant Recruitment

The sample size was calculated using Yamane’s [17] formula. The total sample size was 400, and the participants were recruited between September 1 and 4, 2021. The participants were people who added friends using RamaCovid and met the following inclusion criteria: (1) aged 18 years or older, (2) had used the RamaCovid system at least once, and (3) were willing to participate in the study. The participants were recruited online via the RamaCovid system by broadcasting an infographic about the study, along with a link and quick response (QR) code through which documents were sent to the potential participants. If a person was willing to participate in the study, they selected the “Accept” button. They then filled out 2 questionnaires via Google Forms. The participants who completed both questionnaires were included in the study.

### Instruments and Variables

#### RamaCovid

**Figure 1:**
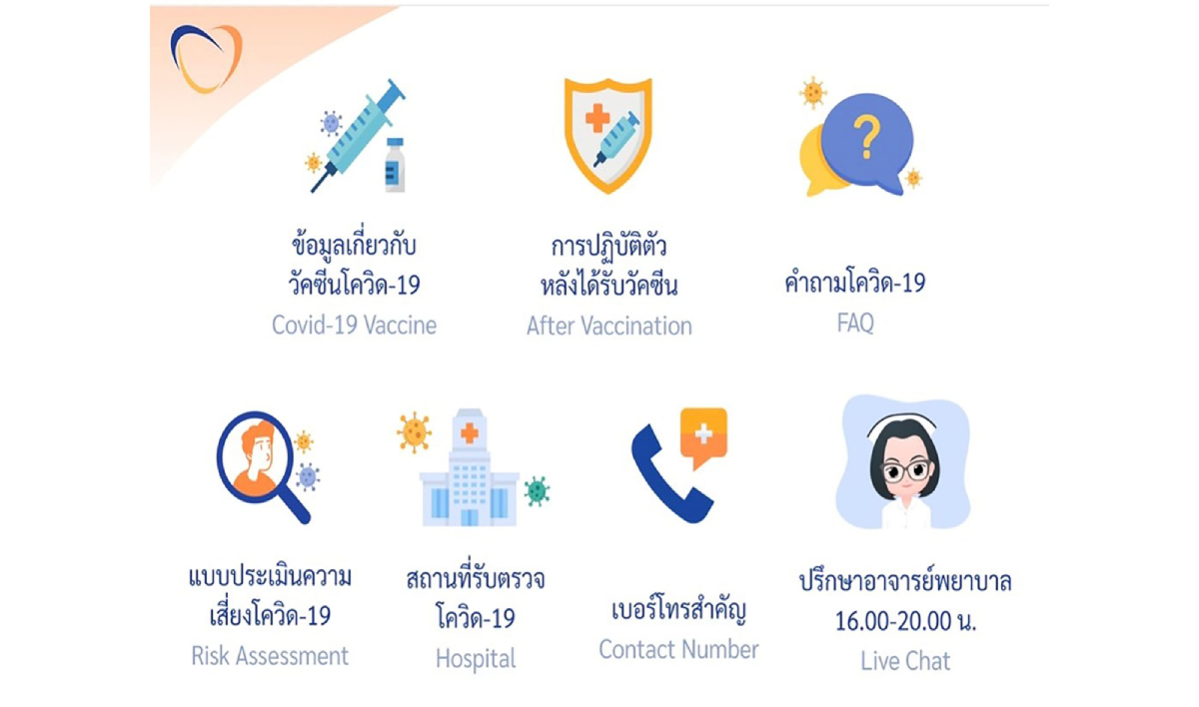
Screenshot of the RamaCovid main menu.

**Figure 2:**
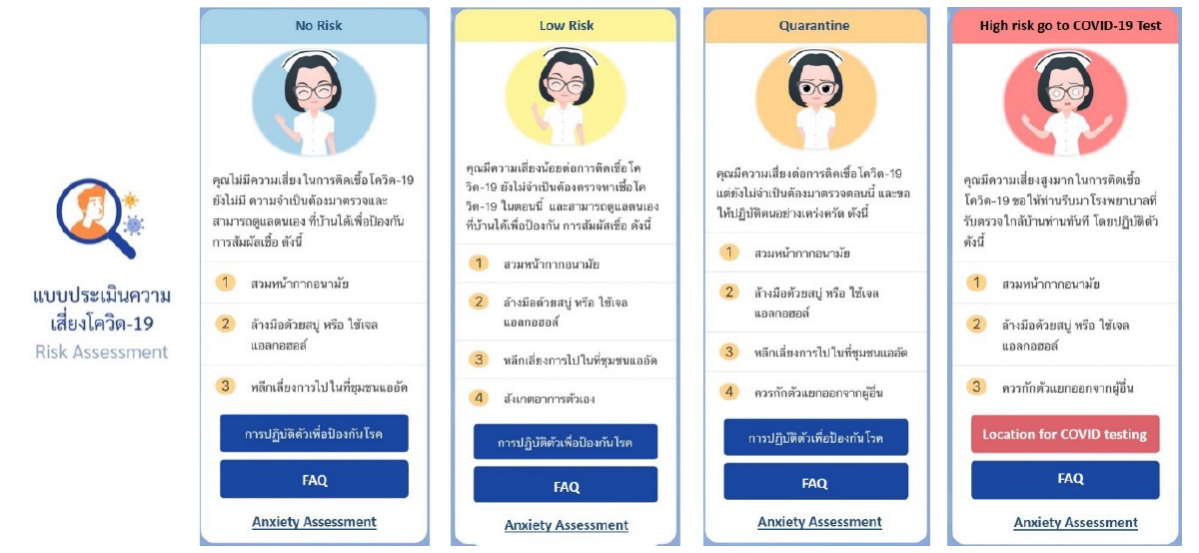
Screenshot of the RamaCovid risk assessment menu.

**Figure 3:**
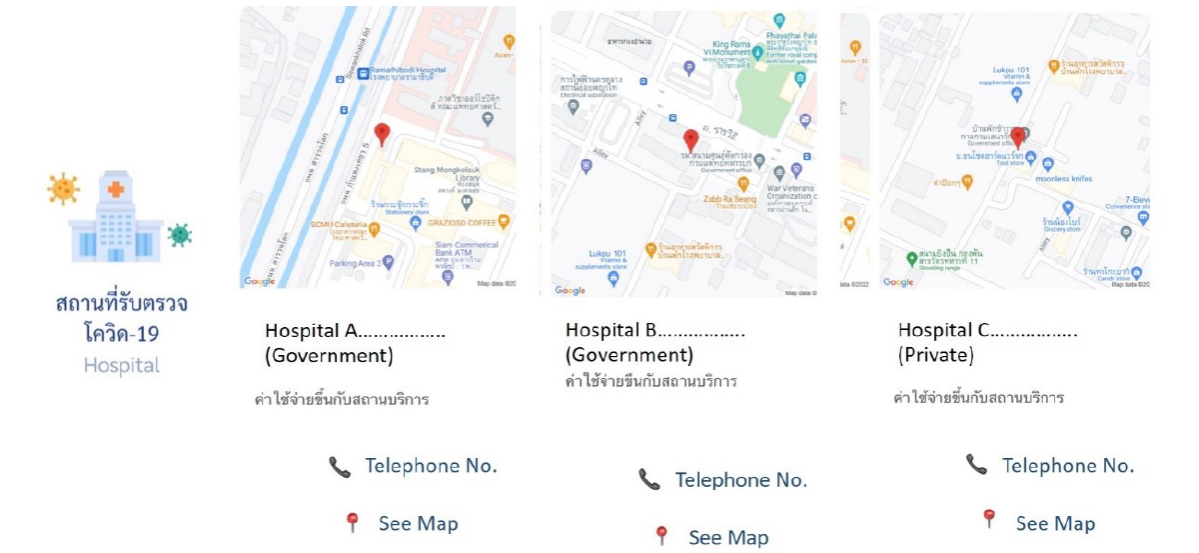
Screenshot of RamaCovid for the location for the RT-PCR test. RT-PCR: reverse transcription polymerase chain reaction.

**Figure 4:**
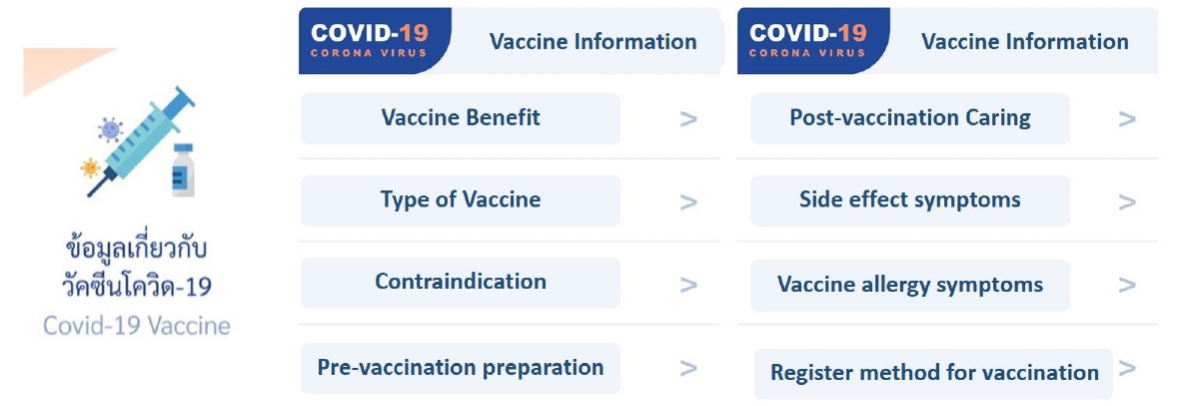
Screenshot of RamaCovid COVID-19 vaccine information.

RamaCovid is a multifaceted mHealth system that guides people to conduct a self-risk assessment and provides COVID-19 education. It consists of 2 functions, a chatbot and a live chat function. The users access the system by choosing the main menu and typing questions or their needs in the textbox. The main menu consists of (1) COVID-19 vaccine information, (2) self-care after vaccination, (3) frequently asked questions, (4) risk assessment, (5) hospital finding, (6) contact number finding, and (7) live chat with health professionals (Figure 1). The chatbot system was developed to provide automatic chat conversations via text messages; it also provides links and infographics. The chatbot can be accessed 24 hours a day. The chatbot is called Somjeed and simulates a conversation with the user. These conversations include information about COVID-19 self-risk assessment, classifying the user’s risk level, and providing advice in accordance with the user’s risk based on guidelines for clinical practice, diagnosis, treatment, and the prevention of health care–associated COVID-19 infection (Figure 2) [11]. Those at high risk or patients under investigation are helped to find the nearest hospital for SARS-CoV-2 RT-PCR testing (Figure 3). Phone numbers are provided for the COVID-19 call center, the emergency hotline, health insurance companies, hospitals, and other health care centers. The chatbot also provides information about COVID-19 characteristics, disease prevention measures, transmission, diagnosis and treatment, quarantine management, self-care measures, and vaccine information (Figure 4). The live chat function is provided for people who have complex questions. When the users choose the live chat on the main menu or type “talk to health professional” in the textbox, the system switches to the live chat function. It is available for 4 hours per day from 4:00 to 8:00 p.m. and is staffed by health professionals. In addition, when there are pressing issues about which people are unsure or when there is an urgent need to disseminate information to the public, we can provide this information via infographics that are broadcast to the system. The system is constantly updated with essential issues and information. For example, vaccine information was added to the system’s main menu when the vaccine program was launched in Thailand.

### Data Collection Instruments

A series of 8 questions was used to collect general information and information about the users’ experience with the RamaCovid system. The following information was collected: age, gender, residential area, education level, history of COVID-19, internet source for seeking health information, how the user became aware of the RamaCovid system, and expectations for RamaCovid. Information about the users’ experience with RamaCovid and the usability of the information provided by RamaCovid was explored through 8 questions with 2 responses (1=ever/useful, 0=never/useless). It covered the following topics: risk assessment, self-care behaviors, finding hospital locations, finding telephone numbers, vaccine knowledge, postvaccination behaviors, frequently asked questions, and the live chat with health professionals.

We developed use and satisfaction questionnaires. The use questionnaire consisted of 10 items that were scored on a 5-point Likert scale. The highest score (5 points) indicated significant use, while the lowest score (1 point) indicated low use. The satisfaction questionnaire consisted of 2 parts. Part 1 comprised 16 items related to the system’s content, appearance, implementation, and overall user satisfaction and were scored using a 5-point Likert scale; the highest score (5 points) indicated very high satisfaction, while the lowest score (1 point) indicated low satisfaction. Part 2 consisted of open-ended questions that solicited the user’s opinions about the problems and obstacles in using the system and suggestions for improvement. Both questionnaires underwent content validity confirmation by 3 experts and had an acceptable level of 1.00. The internal consistency indicated by Cronbach α was .849 for the use questionnaire and .942 for part 1 of the satisfaction questionnaire.

### Ethical Considerations

This study was approved by the Committee on Human Rights Related to Research Involving Human Subjects, Faculty of Medicine, Ramathibodi Hospital, Mahidol University (# COA. MURA2021/265). Before starting the research, the researchers introduced themselves and gave information to the participants about the research objective, data collection, and the benefit the participants would receive. If the participants were willing to participate in this research, the survey system requested the participants to click “Accept” at the bottom instead of signing the informed consent form. The participants could accept, refuse, or withdraw from participate in this research at any time. Their decision did not affect their lives and standard service, and the data were kept confidential and presented in the overall results.

### Statistical Analysis

Descriptive statistics (frequency, percentage, range, mean, and SD) were used to describe the participant characteristics and their use of and satisfaction with the RamaCovid system. The Mann-Whitney *U* test was performed to examine the difference in use and satisfaction between the adult and older adult groups due to the data being not normally distributed. Qualitative data collected from the open-ended questions were categorized into use and satisfaction themes. Each theme was summarized and explained in an overview to support quantitative data.

## Results

### Participant Characteristics

Overall, 400 people completed the online questionnaires. The ages ranged from 18 to 83 years, with a mean age of 46.79 (SD 13.49). Most participants were women (n=273, 68.25%), had a bachelor's degree (n=187, 46.75%), lived in Bangkok (n=200, 50%), and had no history of COVID-19 (n=367, 91.75%). The apps that users most often used to seek health information were Line (n=308, 77%), Facebook (n=283, 70.75%), and YouTube (n=245, 61.25%). The participants became aware of RamaCovid via Icon (n=98, 24.5%), a recommendation from their Line OA (n=73, 18.25%), the School of Nursing website (n=66, 16.5%), and Facebook (n=62, 15.5%). The most prominent expectations for RamaCovid included being provided with self-care knowledge (n=316, 79%), finding a hospital for RT-PCR testing (n=295, 73.75%), and obtaining vaccine information (n=289, 72.25%); see Table 1. The most valuable topics in RamaCovid were information about self-care (n=270, 67.5%), vaccines (n=225, 56.25%), self-risk assessment (n=198, 49.5%), and self-care after vaccination (n=198, 49.5%).

**Table 1:** Participant characteristics and expectations for the RamaCovid system.

Characteristics			Participants (N=400), n (%)
**Age (years): range 18-83 years, mean 46.79, SD 13.49**			
	18-29	47 (11.75)	
	30-39	74 (18.50)	
	40-49	103 (25.75)	
	50-59	97 (24.25)	
	60 and over	79 (19.75)	
**Gender**			
	Men	127 (31.75)	
	Women	273 (68.25)	
**Education**			
	Primary level and lower	12 (3.00)	
	Secondary level and diploma	101 (25.25)	
	Bachelor’s degree	187 (46.75)	
	Postgraduate	100 (25.00)	
**Residential area**			
	Bangkok	200 (50.00)	
	Central	108 (27.00)	
	West	34 (8.50)	
	Northeast	17 (4.25)	
	East	16 (4.00)	
	North	14 (3.50)	
	South	11 (2.75)	
**History of COVID-19**			
	No	367 (91.75)	
	Yes	33 (8.25)	
**Source for seeking health information**			
	Google	332 (83.00)	
	YouTube	245 (61.25)	
	Line	308 (77.00)	
	Facebook	283 (70.75)	
	School of Nursing website	214 (53.50)	
	Instagram	49 (12.25)	
	Twitter	47 (11.75)	
	Other (friends/staff/book)	17 (4.25)	
**How one became aware of RamaCovid**			
	Icon	98 (24.50)	
	Official Line account menu	73 (18.25)	
	School of Nursing website	66 (16.50)	
	Facebook	62 (15.50)	
	QR^a^ code	34 (8.50)	
	Timeline	29 (7.25)	
	Friends	29 (7.25)	
	Other	9 (2.25)	
**Expectations for RamaCovid**			
	Self-care	316 (79.00)	
	Find location for RT-PCR^b^ testing	295 (73.75)	
	Vaccine information	289 (72.25)	
	Self-risk assessment	252 (63.00)	
	Monitor situation	171 (42.75)	
	Live chat with health professional	156 (39.00)	
	Obtain test results	112 (28.00)	
**Most useful topics in RamaCovid**			
	Self-care	270 (67.50)	
	Vaccine information	225 (56.25)	
	Self-care after vaccination	198 (49.50)	
	Self-risk assessment	198 (49.50)	
	Find telephone numbers	128 (32.00)	
	Find location for RT-PCR testing	125 (31.25)	
	Live chat with health professionals	112 (28.00)	
	Frequently asked questions	73 (18.25)	

^a^QR: quick response

^b^RT-PCR: reverse transcription polymerase chain reaction.

The most used RamaCovid menu selections were to obtain information about the vaccines (n=254, 63.5%), self-care knowledge (n=248, 62%), and self-care after vaccination (n=222, 55.5%). The users who accessed RamaCovid had high satisfaction in each area (mean 4.15-4.49, SD 0.62-0.8). The highest mean satisfaction scores were for users who engaged in a live chat with a health professional, sought information about self-care after vaccination, or sought telephone numbers. Considering the age group, the adult group had a higher satisfaction in their experience of using the RamaCovid menu compared to the older adult group (Table 2).

**Table 2:** Experience (satisfaction) of using the RamaCovid system.

RamaCovid menu	Overall (N=400)		Adults (18-59 years, n=321)			Older adults (60 years and over, n=79)	
	n (%)	Mean (SD)	n (%)	Mean (SD)	n (%)		Mean (SD)
Vaccine information	254 (63.50)	4.24 (0.68)	203 (63.24)	4.26 (0.70)	51 (64.56)		4.18 (0.59)
Self-care knowledge	248 (62.00)	4.18 (0.66)	190 (59.19)	4.24 (0.65)	58 (73.42)		3.97 (0.65)
Self-care after vaccination	222 (55.50)	4.34 (0.62)	174 (54.21)	4.36 (0.59)	48 (60.76)		4.27 (0.71)
Self-risk assessment	208 (52.00)	4.21 (0.70)	167 (52.02)	4.22 (0.71)	41 (51.90)		4.20 (0.64)
Find location for RT-PCR^a^ testing	129 (32.25)	4.15 (0.70)	103 (32.09)	4.19 (0.67)	26 (32.91)		3.96 (0.77)
Find telephone numbers	124 (31.00)	4.25 (0.80)	99 (30.84)	4.31 (0.79)	25 (31.65)		4.00 (0.82)
Frequently asked questions	117 (29.25)	4.18 (0.66)	94 (29.28)	4.32 (0.66)	23 (29.11)		3.91 (0.60)
Live chat with health professionals	86 (21.50)	4.49 (0.78)	64 (19.94)	4.58 (0.77)	22 (27.85)		4.23 (0.75)

^a^RT-PCR: reverse transcription polymerase chain reaction.

### Use of and Satisfaction With RamaCovid

**Figure 5:**
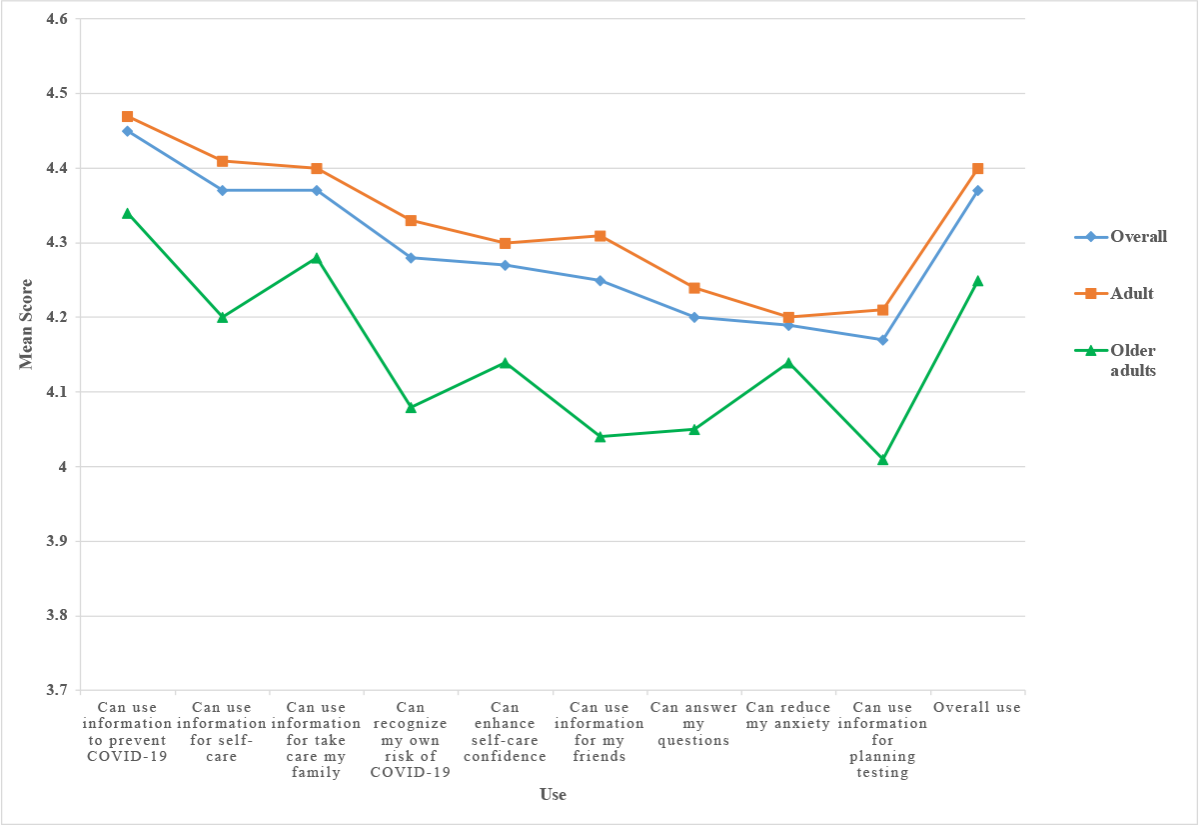
Use of the RamaCovid system.

RamaCovid provides COVID-19 information to users. The people who used RamaCovid had high use (mean 4.37, SD 0.63) and satisfaction (mean 4.30, SD 0.70) scores. The top 3 use areas were ways to prevent COVID-19 (mean 4.45, SD 0.63), self-care information (mean 4.37, SD 0.64), and information about taking care of the family (mean 4.37, SD 0.67). The 3 lowest-use areas were information about RT-PCR testing, releasing anxiety, and looking at frequently asked questions. The adult group had a higher average score of use than the older adult group in all topics (Figure 5). The results showed a significant difference in use between adults and older adults in self-care information (*P*=.014), recognizing my own risk (*P*=.002), enhancing self-care confidence (*P*=.031), using information for my friends (*P*=.001), using information to answer my questions (*P*=.029), and using information for planning testing (*P*=.015); see Table 3.

**Table 3:** Use (Mann-Whitney *U* test) of the RamaCovid system (N=400).

Topics and age group		Mean (SD)	Mean rank	Sum of ranks
**Can use information to prevent COVID-19 (*P*=.065)**				
	Overall	4.45 (0.68)	N/A^a^	N/A
	Adults	4.47 (0.63)	205.20	65,868.50
	Older adults	4.34 (0.62)	181.41	14,331.50
**Can use information for self-care (*P*=.014)**				
	Overall	4.37 (0.68)	N/A	N/A
	Adults	4.41 (0.62)	206.77	66,372.50
	Older adults	4.20 (0.69)	175.03	13,827.50
**Can use information for take care my family (*P*=.194)**				
	Overall	4.37 (0.68)	N/A	N/A
	Adults	4.40 (0.66)	203.84	65,432.50
	Older adults	4.28 (0.73)	186.93	14,767.50
**Can recognize my own risk of COVID-19 (*P*=.002)**				
	Overall	4.28 (0.68)	N/A	N/A
	Adults	4.33 (0.65)	208.55	66,945.00
	Older adults	4.08 (0.66)	167.78	13,255.00
**Can enhance self-care confidence (*P*=.031)**				
	Overall	4.27 (0.68)	N/A	N/A
	Adults	4.30 (0.69)	206.09	66,155.00
	Older adults	4.14 (0.64)	177.78	14,045.00
**Can use information for my friends (*P*=.001)**				
	Overall	4.25 (0.68)	N/A	N/A
	Adults	4.31 (0.72)	209.32	67,191.50
	Older adults	4.04 (0.67)	164.66	13,008.50
**Can answer my questions (*P*=.029)**				
	Overall	4.20 (0.68)	N/A	N/A
	Adults	4.24 (0.73)	206.26	66210.50
	Older adults	4.05 (0.70)	177.08	13989.50
**Can reduce my anxiety (*P*=.378)**				
	Overall	4.19 (0.68)	N/A	N/A
	Adults	4.20 (0.76)	202.82	65,106.50
	Older adults	4.14 (0.71)	191.06	15,093.50
**Can use information for planning testing (*P*=.015)**				
	Overall	4.17 (0.68)	N/A	N/A
	Adults	4.21 (0.76)	206.87	66,406.00
	Older adults	4.01 (0.67)	174.61	13,794.00
**Overall use (*P*=.057)**				
	Overall	4.37 (0.68)	N/A	N/A
	Adults	4.40 (0.63)	205.39	65,930.50
	Older adults	4.25 (0.63)	180.63	14,269.50

^a^N/A: not applicable.

**Figure 6:**
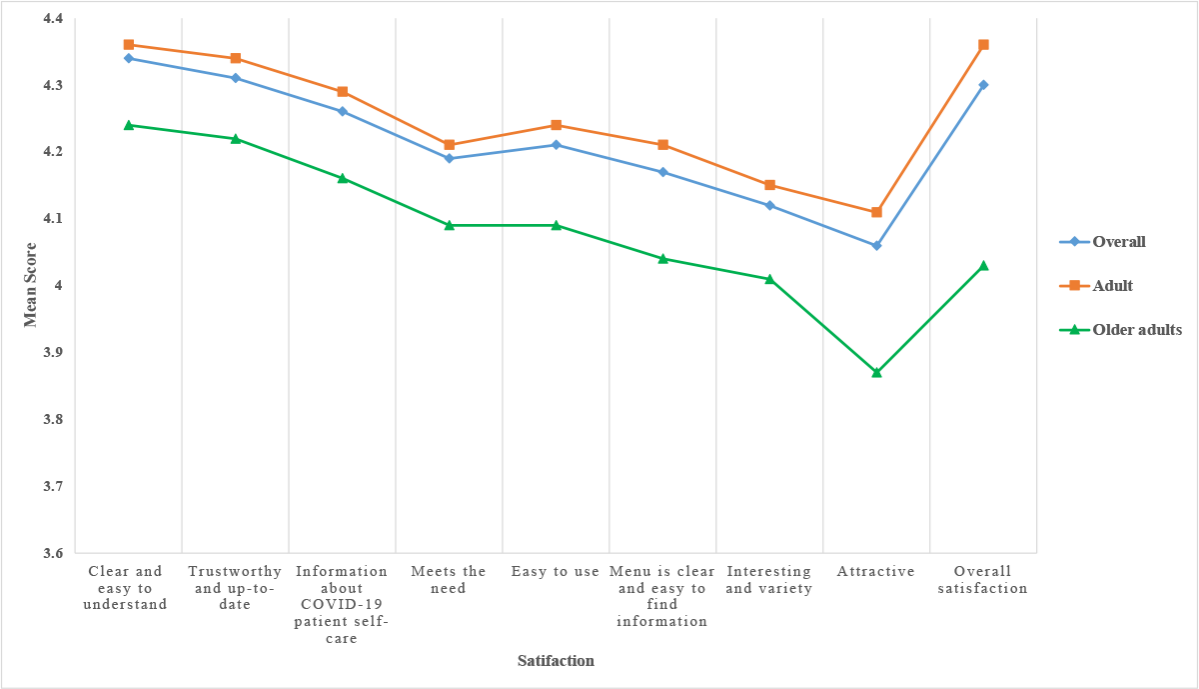
Satisfaction with the RamaCovid system.

The 3 satisfaction areas with the highest scores were the information being clear and easy to understand (mean 4.34, SD 0.68), the information being trustworthy and up to date (mean 4.31, SD 0.68), and the information about COVID-19 patient self-care (mean 4.26, SD 0.68). The lowest satisfaction scores were for the information provided being attractive, interesting, having variety, and being easy to use. The adult group had a higher average score of satisfaction than the older adult group in all topics (Figure 6). The results showed a significant difference in satisfaction between adults and older adults in attractiveness (*P*=.007) and overall satisfaction (*P*<.001); see Table 4.

**Table 4:** Satisfaction (Mann-Whitney *U* test) with the RamaCovid system (N=400).

Topics and age group		Mean (SD)	Mean rank	Sum of ranks
**Clear and easy to understand (*P*=.085)**				
	Overall	4.34 (0.68)	N/A^a^	N/A
	Adults	4.36 (0.65)	204.93	65,784.00
	Older adults	4.24 (0.63)	182.48	14,416.00
**Trustworthy and up to date (*P*=.10)**				
	Overall	4.31 (0.68)	N/A	N/A
	Adults	4.34 (0.68)	204.75	65,726.00
	Older adults	4.22 (0.65)	183.22	14,474.00
**Information about COVID-19 patient self-care (*P*=.087)**				
	Overall	4.26 (0.68)	N/A	N/A
	Adults	4.29 (0.70)	204.93	65,783.00
	Older adults	4.16 (0.63)	182.49	14,417.00
**Meets the need (*P*=.126)**				
	Overall	4.19 (0.68)	N/A	N/A
	Adults	4.21 (0.70)	204.47	65,635.50
	Older adults	4.09 (0.70)	184.36	14,564.50
**Easy to use (*P*=.111)**				
	Overall	4.21 (0.68)	N/A	N/A
	Adults	4.24 (0.73)	205.58	65,992.00
	Older adults	4.09 (0.68)	179.85	14,208.00
**Clear menu and easy-to-find information (*P*=.051)**				
	Overall	4.17 (0.68)	N/A	N/A
	Adults	4.21 (0.73)	205.60	65,997.00
	Older adults	4.04 (0.72)	179.78	14,203.00
**Interesting, with variety (*P*=.111)**				
	Overall	4.12 (0.68)	N/A	N/A
	Adults	4.15 (0.77)	204.71	65,712.00
	Older adults	4.01 (0.73)	183.39	14,488.00
**Attractiveness (*P*=.007)**				
	Overall	4.06 (0.68)	N/A	N/A
	Adults	4.11 (0.75)	207.59	66,636.00
	Older adults	3.87 (0.71)	171.70	13,564.00
**Overall satisfaction (*P*<.001)**				
	Overall	4.30 (0.68)	N/A	N/A
	Adults	4.36 (0.69)	211.88	68,014.00
	Older adults	4.03 (0.62)	154.25	12,186.00

^a^N/A: not applicable.

### Users’ Opinions and Suggestions for RamaCovid

The opinions provided by the users indicated that RamaCovid provides a benefit to them. The users could access COVID-19 information about self-risk assessment and self-care and how to prevent COVID-19. RamaCovid was an additional channel for accessing up-to-date information. Furthermore, it was easier to access than the call center, especially during peak times. The live chat feature was a favorite functionality because it gave people the information they needed and lowered their stress and anxiety. The users valued the expertise of the staff and suggested extending the service time for the live chat. The self-risk assessment system helped the user decide whether to go to the hospital; thus, it reduced travel and congestion in hospitals. Up-to-date information was a critical issue for the users, and there should be continual improvement and updates as the COVID-19 pandemic unfolds. Users wanted to know the most up-to-date information at both national and international levels. They also wanted the system to be linked to the vaccination reservation system and to expand to other common diseases. Making the information attractive and interesting is a challenge for system improvement. For example, users recommended cartoon animations to increase the system’s attractiveness and attract children and adolescents’ attention.

## Discussion

### Principal Results

This study found that many older adults could access the RamaCovid system. Compared to similar previous studies, more participants were young and middle-aged adults [18,19]. The oldest study participant was 83 years old; however, only 1 in 5 RamaCovid users was aged 60 years or over. Nevertheless, this finding implies that older adults can use RamaCovid. However, previous studies have shown that older adults can have limitations in accessing mHealth, such as reduced willingness, a lack of internet connection, and low information technology literacy [20-23]. A previous study found that government and family support could empower older adults to increase their accessibility to mHealth, which improved their health during the COVID-19 pandemic [21]. Thus, preparing for this support should be a concern in anticipation of future disease pandemics.

The results revealed that RamaCovid can meet users' expectations, and the system received positive use and satisfaction evaluations. These results indicated the successful implementation of RamaCovid. These findings align with many previous studies from Indonesia, the Netherlands, South Korea, and Saudi Arabia, where mHealth apps have been accepted and used by citizens. These apps have effectively managed services and delivered health information during the pandemic [24-28]. In addition, the findings are consistent with previous studies that demonstrated the usefulness of mHealth apps during the COVID-19 pandemic in the general population [29] and health professionals [14]. The success of RamaCovid might be because it is a multifaceted system that includes risk assessment, hospital GPS location information, COVID-19 education, updated vaccine information, and the ability to live-chat with a health professional. In particular, the call center experiences helped us design specific functions to meet users' needs.

The automatic chatbot was designed by translating information into text, videos, and infographics, with the aim of helping people more easily understand the information. The GPS location and telephone numbers of nearby hospitals were also provided to help people find a nearby hospital for RT-PCR testing. These functions may have led to high use and satisfaction. This study revealed that the RamaCovid system can provide health information and is an alternative way to work with the call center during a pandemic crisis.

The findings showed that the live chat function is vital for communicating with experts and health professionals, and this function had the highest satisfaction among experienced users. Moreover, the information and opinions provided by the participants indicated that the live chat function was able to address their concerns and helped reduce their anxiety. They recommended expanding the service time of the live chat function. The findings revealed that users with complicated problems or anxiety needed to communicate with a health professional directly, which is in accordance with previous studies that showed communicating with a health professional could decrease one’s anxiety [30,31]. Thus, a live chat function should be seriously considered when developing mHealth systems in future pandemics.

Although older adults had high use and satisfaction with RamaCovid, they had average scores lower than the adult group. The reason may be that older adults have more expectation for the system. RamaCovid provides self-risk assessment and health education; however, it cannot support hospital services, such as transportation, hospital admission, and vaccination reservation. For example, although older adults could find the location for COVID-19 testing from the system, they could not go to the hospital by themselves and needed the transportation system to take them to the hospital. Moreover, during the crisis situation, older adults got a COVID-19 infection at that time and they wanted to find a hospital for admission. These reasons may affect the use of the RamaCovid system in older adults in terms of self-care, self-care confidence, answering their questions, and information for planning testing. The self-risk assessment function needs the information before classifying the user’s risk level. Previous studies have shown that privacy and confidentiality are barriers to using mHealth in older adults [32]; hence, older adults may be concerned about the privacy and confidentiality of their information and they did not continue to complete the form and get the result.

Regarding satisfaction, older adults had lower satisfaction than adults, especially with regard to attractiveness. The reason may be that older adults may be unfamiliar with the function of RamaCovid, which is a different platform than what they used. In addition, older adults may have physical limitations, such as a decrease in visual acuity, effecting reading and typing ability. Some chatbot responses of this system were provided in text messages, which contributed to the difficulty in reading and understanding for older adults. The new platform and physical limitation were reported in previous studies as barriers influencing satisfaction in older adults [33].

The system's credibility increased people's trust in RamaCovid and made it more likely that they would follow the advice. The results showed that users accepted the information from the system because they perceived it as trustworthy and up to date. Accurate and up-to-date information is critical when developing an mHealth system.

Although RamaCovid provides information about COVID-19 and self-risk assessment, the users advocated for a more comprehensive 1-stop service for COVID-19 issues via the system. For example, the users suggested that RamaCovid be integrated with the vaccination reservation system and be able to help people with COVID-19 find a hospital for admission. These functional deficits are the limitations of this system. Thus, a 1-stop service system is a remaining challenge for the next step in the pandemic response. The system could also be improved with attractive and interesting media to increase user satisfaction and broaden the accessibility to all age groups.

### Limitations

This study included people who can access a mobile phone**,** and most participants had a high education level. Thus, the sample does not represent the general Thai population, and generalizability is limited. Additionally, this system is not provided in the English language for foreigners in Thailand and voice messages for people with disabilities.

### Future Research

Further research should determine the effect of this system on knowledge and health literacy in this population. In addition, a 1-stop service app, an English version, and voice messages should be developed for the next step in the pandemic response or other situations. mHealth for patients with noncommunicable diseases should be developed to provide self-care information using a chatbot system and health counseling with experts and health professionals via live chat in the long run.

### Conclusion

This study investigated the multifaceted RamaCovid mHealth system and found that it has been beneficial for communicating with the public during the COVID-19 pandemic. It was an alternative way to work with the call center and increased access to health information and health care services. People can use the information obtained from the system to take care of themselves and their families. The live chat function allowed direct communication with health professionals, reducing the users’ anxiety. Providing ongoing updated information, improving the attractiveness of the media information, and considering age group differences are important issues for further system development.

## Abbreviations

GPS: Global Positioning System
Line OA: Line official account
mHealth: mobile health
QR: quick response
RT-PCR: reverse transcription polymerase chain reaction

## Data Availability

The data sets generated and analyzed during the study are available from the corresponding author upon reasonable request.
